# Comment on “Effects of Arsenite during Fetal Development on Energy Metabolism and Susceptibility to Diet-Induced Fatty Liver Diseases in Male Mice” and “Mechanisms Underlying Latent Disease Risk Associated with Early-Life Arsenic Exposure: Current Trends and Scientific Gaps”

**DOI:** 10.1289/ehp.1611345

**Published:** 2016-06-01

**Authors:** Paul A. Fowler, Amanda J. Drake, Peter J. O’Shaughnessy, Siladitya Bhattacharya, Andrea Raab, Kevin D. Sinclair, Jörg Feldmann, Andrew A. Meharg

**Affiliations:** 1 Institute of Medical Sciences, University of Aberdeen, Aberdeen, United Kingdom; 2 University/BHF Centre for Cardiovascular Science, University of Edinburgh, Queen’s Medical Research Institute, Edinburgh, United Kingdom; 3 Institute of Biodiversity, Animal Health and Comparative Medicine, College of Medical, Veterinary and Life Sciences, University of Glasgow, Glasgow, United Kingdom; 4 Institute of Applied Health Sciences, University of Aberdeen, Aberdeen, United Kingdom; 5 TESLA (Trace Element Speciation Laboratory) and Marine Biodiscovery Laboratory, University of Aberdeen, Aberdeen, United Kingdom; 6 School of Biosciences, Sutton Bonington Campus, University of Nottingham, Loughborough, United Kingdom; 7 Institute for Global Food Security, Queen’s University Belfast, Belfast, United Kingdom

We were very interested to read the excellent article and commentary on arsenic and health, with respect to prenatal exposures, metabolic health, and underlying mechanisms, by Ditzel et al. and Bailey et al. For many years there have been growing concerns as knowledge accumulates about the long-term consequences of early-life influences on adult health (Fleming et al. 2015), including environmental programming resulting from prenatal exposure to arsenic (Farzan et al. 2013). These concerns rightly extend to metabolic consequences for the offspring (Ashley-Martin et al. 2015) and also encompass epigenetic modifications as a result of developmental exposures to arsenic and other metals (Bailey and Fry 2014).

The elephant in the room, however, is the paucity of knowledge about what is happening to the human fetus early in gestation. A great deal of emphasis is placed on levels of toxicants in maternal urine or plasma, and on levels in the offspring at term (usually in cord blood), which is then often mislabeled as “fetal.”

Last year we published a study of 55 normally progressing, electively terminated second-trimester human fetuses where changes in fetal hepatic cobalt levels were traced through vitamin B_12_ to changes in one-carbon metabolism and alterations in DNA methylation (Drake et al. 2015). We also characterized hepatic concentrations of essential and nonessential elements. Maternal smoking was validated by measuring circulating fetal cotinine, and we were surprised to find no increase in the number of fetal livers with cadmium levels above limits of detection when the mother smoked (only 5 of 55 fetuses had hepatic levels of cadmium above limits of detection, irrespective of smoke exposure) (Drake et al. 2015), despite a known increase in maternal cadmium levels with smoking (Sikorski et al. 1988). This clearly demonstrates the importance of studying fetal toxicant burden in our own species rather than simply making assumptions from animal models.

Relevant to Ditzel et al. and Bailey et al., we also published fetal hepatic arsenic concentrations and found levels above limits of detection in 39 of 55 fetuses, with a median arsenic concentration of 39.8 ng/g dry liver weight (Drake et al. 2015). As with cadmium, maternal smoking did not significantly alter fetal hepatic levels of arsenic, and arsenic concentrations did not change between 11 and 21 weeks of gestation. These kinds of “real-life” human fetal data are essential to improve our understanding of the risks to the developing human, the points during gestation when those risks are most acute, and the key underlying mechanisms.

*Editor’s note: In accordance with journal policy, Ditzel et al. and Fry et al. were asked whether they wanted to respond to this letter. Neither group chose to do so.*
